# Selection and Validation of the Most Suitable Reference Genes for Quantitative Real-Time PCR Normalization in *Salvia rosmarinus* under In Vitro Conditions

**DOI:** 10.3390/plants11212878

**Published:** 2022-10-27

**Authors:** Rohit Bharati, Madhab Kumar Sen, Ram Kumar, Aayushi Gupta, Vishma Pratap Sur, Ingrid Melnikovová, Eloy Fernández-Cusimamani

**Affiliations:** 1 Department of Crop Sciences and Agroforestry, The Faculty of Tropical AgriSciences, Czech University of Life Sciences Prague, Kamýcká 129, 165 00 Prague 6, Czech Republic; 2 Department of Agroecology and Crop Production, Faculty of Agrobiology, Food and Natural Resources, Czech University of Life Sciences Prague, Kamýcká 129, 165 00 Prague 6, Czech Republic; 3 Department of Plant Protection, Faculty of Agrobiology, Food and Natural Resources, Czech University of Life Sciences Prague, Kamýcká 129, 165 00 Prague 6, Czech Republic; 4 Department of Botany and Plant Physiology, Faculty of Agrobiology, Food and Natural Resources, Czech University of Life Sciences Prague, Kamýcká 129, 165 00 Prague 6, Czech Republic; 5 Laboratory of Reproductive Biology, Institute of Biotechnology of the Czech Academy of Sciences, BIOCEV, Prumyslova 595, 252 50 Vestec, Czech Republic

**Keywords:** housekeeping genes, plant tissue culture, nanoparticle, real-time PCR, rosemary, secondary metabolite, reference genes

## Abstract

*Salvia rosmarinus* L. (rosemary) is known to have a wide range of pharmacological effects including antidiabetic, anticarcinogenic, and antitumorigenic properties owing to its secondary metabolites. Studies aiming to elevate these metabolites have utilized various elicitors and stresses under in vitro conditions, although underlying molecular mechanisms remain unexplored. Gene expression studies using RT-qPCR might provide valuable information regarding how plant and plant cells interact and perceive various treatments and elicitors. However, despite being able to calculate accurate fold changes, the accuracy of the RT-qPCR data highly depends on the expression of reference genes. To the best of our knowledge, there is no information available on the stable reference genes in rosemary under in vitro conditions. Thus, in this paper, we assessed the stability of seven commonly used reference genes under different elicitor and stress conditions using RT-qPCR. Thereafter, the five most commonly used software and algorithms (comparative ΔCt, BestKeeper, NormFinder, geNorm, and RefFinder) were used to rank the candidates based on their expression stabilities. In conclusion, we recommend using a combination of *F1-ATPase*, *ATP synthase* and *ACCase* to normalize the gene expression experiments in rosemary under in vitro conditions. The selected reference genes were verified using *4-coumarate-CoA ligase*, a pharmacologically important gene, whose expression might alter under nanoparticle treatment. Additionally, reference genes for several plant tissues, elicitors, and stresses are also proposed. The conclusions obtained from this current study will accelerate the future molecular work in *S. rosmarinus* and other related species.

## 1. Introduction

Rosemary (*Salvia rosmarinus* L.), belonging to the Lamiaceae family, is an aromatic plant with well-known pharmacological effects. The wide range of pharmacological effects of this perennial herb is mainly due to its ample secondary metabolites, which are known to have antioxidant, antibacterial, anti-inflammatory, antidiabetic, anticarcinogenic, and antitumorigenic activities [1,2]. In addition to these, recent studies have also shown that carnosic acid, carnosol, and rosmanol (phytochemicals present in rosemary) have inhibitory effects against the coronavirus’s main protease (SARS-CoV-2 M^pro^) [3,4]. Consequently, interest has been raised towards increasing these pharmacologically important secondary metabolites of this species across industries. In this scenario, plant tissue culture technology can be an excellent option for large-scale disease-free plant and tissue production with elevated quantities of secondary metabolites from rosemary and other medicinal plants, irrespective of their growing seasons. Furthermore, the treatment of various elicitors, such as nanoparticles (NPs) and others in culture systems, can also boost or change the secondary metabolite level [5]. Even though studies aiming to increase the secondary metabolites through plant tissue culture in rosemary are constantly in process, many more will be needed in the future to accomplish its demands in industrial production. In order to effectively manipulate the synthesis of these metabolites under in vitro conditions, underlying molecular mechanisms needs to be explored. Currently, based on the previous literature studies, there is limited information available about the genes and metabolic pathways in rosemary [6]. Hence, gene expression analysis using quantitative real-time PCR (RT-qPCR) can be an essential tool to obtain insights into the metabolic pathways of secondary metabolites in specific tissues or under different conditions.

Owing to its high specificity, rapidity, and sensitivity, RT-qPCR has gained an edge over the traditional polymerase chain reaction (PCR) for comparative expression studies. Although RT-qPCR can calculate accurate fold changes, its accuracy is extremely reliant on the expression of a suitable reference gene [7]. The steps of RT-qPCR are prone to technical noises and variations in the sample preparation. Hence, to nullify these variations, appropriate normalization methods are necessary [8]. The target gene transcription levels must be normalized with suitable reference gene transcription levels. Any inaccuracies in selecting a suitable reference gene may lead to deceptive results [9,10]. The most commonly used references genes in plant gene expression studies include *ubiquitin (UBQ), eukaryotic elongation factor (eEF), α-tubulin (α-TUB), β-tubulin (β-TUB), actin (ACT), ribosomal RNA genes (rRNA), glyceraldehyde-3-phosphate dehydrogenase (GAPDH), Acetyl CoA Carboxylase (ACCase)*, etc. [7,8]. Although it can be assumed that these genes will have a stable expression in any given condition, numerous studies have provided evidence that their expression levels can vary considerably across plant species, stress conditions, or even developmental stages [7,11].

Interest in plant tissue culture or in vitro plant culture has grown mainly due to its promising ability to produce improved crop varieties and a high yield of crucial secondary metabolites. Several efforts have been made to enhance the production of important secondary metabolites using different biotic and abiotic factors. Currently, the addition of elicitors such as nanoparticles has gained worldwide interest owing to their success in enhancing secondary metabolites in many species. A recent study reported that silver nanoparticle treatment could boost the carnosic acid content in *Rosmarinus officinalis* L. [12]. The rising interest in using nanoparticles in commercially important medicinal plants (such as rosemary) will increase the need for gene expression studies in these species. To the best of our knowledge, there are no reports on the identification of the most suitable reference genes for gene expression studies under abiotic stress in rosemary produced under in vitro conditions. Hence, this study aims to identify a suitable reference gene for gene expression studies in in vitro *S. rosmarinus*. We selected and identified seven common candidate reference genes (*18S rRNA, 25S rRNA, 28S rRNA, ACCase, GAPDH, ATP-synthase,* and *F1-ATPase*) from previous literature studies [7,8,13,14]. The genes identified were from *S. rosmarinus*, related or other plant species depending on their homology, and how frequently are utilized. Further, these genes were assessed for their gene expression stability in three different plant tissues/organs (callus, stem, and leaf), temperature stress (heat stress and cold stress), two elicitor stress (casein hydrolysate and jasmonic acid), osmotic stress (sorbitol) and salt stress (NaCl). Five different widely used statistical algorithms for reference gene analysis (comparative ΔCt [15], BestKeeper [16], NormFinder [17], geNorm [18], and RefFinder [19]) were used to identify the best candidate. To validate the reliability of the most stable reference gene, relative expression levels of a target gene (*4-coumarate-CoA ligase* (*4CL*)) was analyzed under an NP stress experiment. *4CL* catalyzes the enzymatic reactions in the general phenylpropanoid pathway and participates in the synthesis of flavonoids. All flavonoids are produced from one molecule of p-coumaroyl-CoA and three molecules of malonyl-CoA. Flavonoids are pharmacologically important compounds with numerous probable medicinal properties, including anticancer, antioxidant, anti-inflammatory, and anti-inflammatory activities [20]. Additionally, suitable candidates under each mentioned experimental condition were also identified. Our study will provide a basis for current and future gene expression studies in *S. rosmarinus* and its related species and will assist in forthcoming molecular studies aiming for a better understanding of the metabolic pathways associated with secondary metabolites.

## 2. Results

### 2.1. Callus Induction and Plant Regeneration

**Figure 1:**
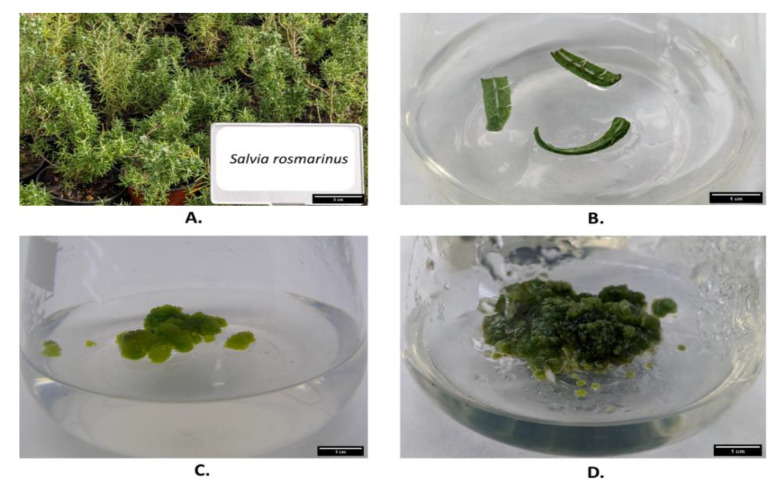
In vitro culture of *Salvia rosmarinus* L: (**A**) Source of the explant. The explants were collected from the well-maintained botanical garden of the Faculty of Tropical AgriSciences, Czech University of Life Sciences, Prague; (**B**) The leaves were used as explants and transferred to MS medium (C1); (**C**) Callus after 10 days and (**D**) Callus after 20 days.

Nodal segments of *S. rosmarinus* subjected to basic MS without any phytohormones were sufficient to establish and propagate under in vitro conditions. For callus induction, all the studied combinations of phytohormones were able to induce callus using leaf segment as an explant after 2 weeks (Table 1 and Figure 1). Despite all combinations being successful in inducing callus, healthy calli with a faster proliferation rate were generated on media C1 when compared to other combinations. The generated calli on C1 were light green to dark green in color, structurally friable, and loosely packed (Figure 1). Hence, they were selected for further treatment. The calli were sub-cultured every 30 days.

### 2.2. Primer Efficiency and Candidate Genes Expression

**Figure 2:**
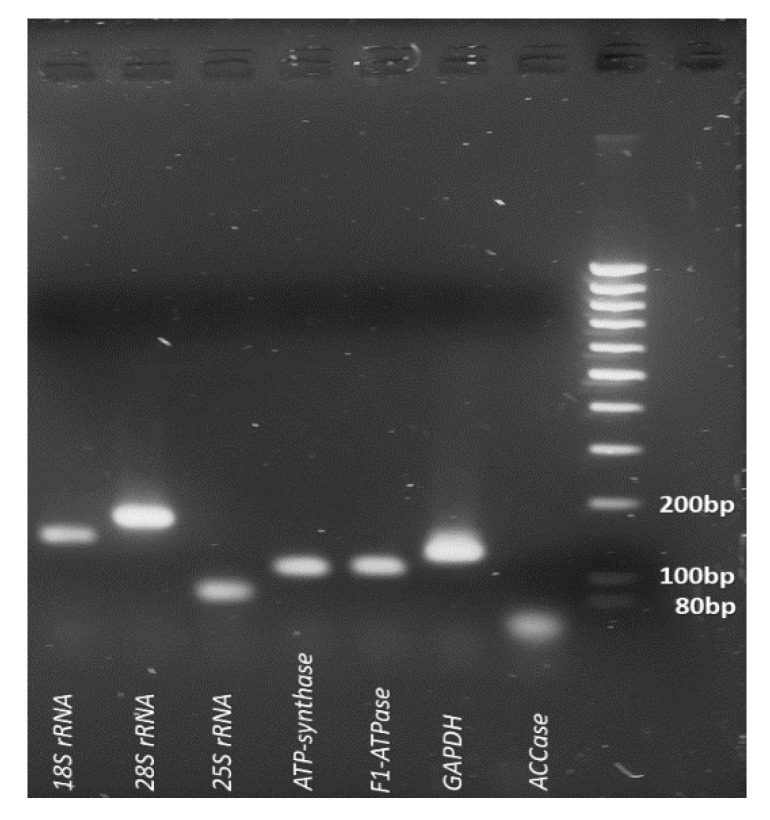
Gel picture showing single bands for each candidate reference gene. The PCR amplicons were loaded on 1.8% agarose gel.

**Figure 3:**
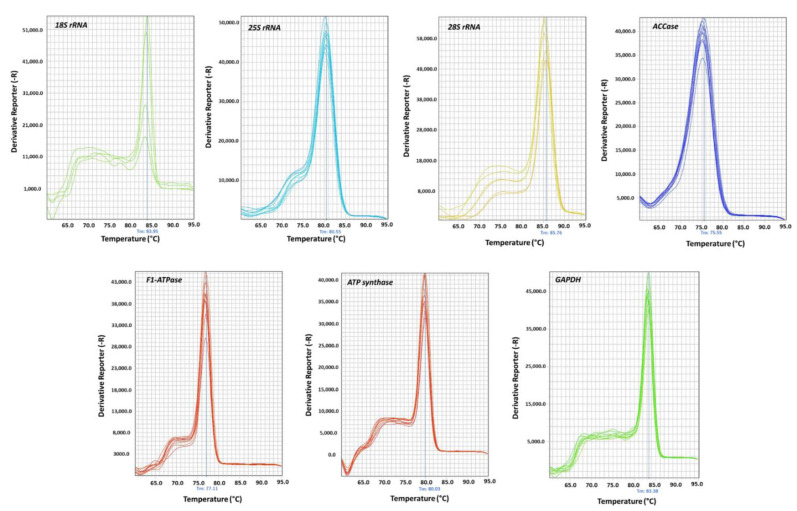
Melting curve analysis of each candidate reference genes.

**Figure 4:**
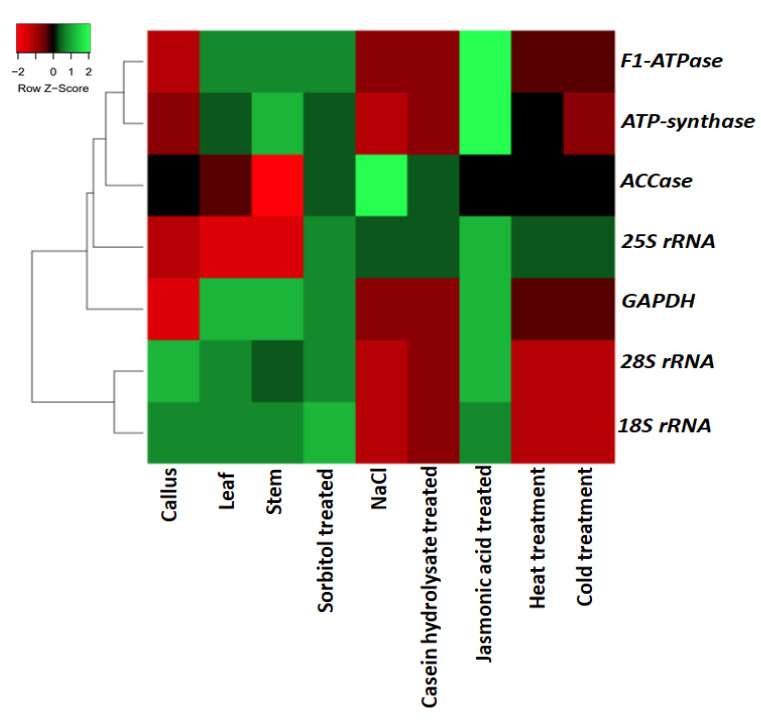
Heat map of candidate reference genes based on the mean Ct values.

The quality and the quantity of the extracted RNA was checked using 1.2% agarose gel electrophoresis and NanoDrop spectrophotometer (NanoDrop™ 2000/2000c Spectrophotometer, Thermo Scientific™, Waltham, MA, USA), respectively. A260/A280 values ranged from 1.90 to 1.96 for all the samples. A total of 1000ng of each RNA sample was further used to synthesize the cDNA. Thereafter, the designed primers were tested in general PCR using diluted cDNA samples. All the amplicons produced from the primers showed a single band in 1.8% agarose gel (Figure 2). In all the RT-qPCR experiments, a single peak was obtained for all the primers (Figure 3). Their efficiency values and correlation coefficient values ranged between 93.87% to 104.69% and 0.9823 to 0.9997, respectively (Table S1). The expression profile of the eight nominees under distinct experimental conditions is shown in Figure 4. Among the seven candidate genes, *18S rRNA* (mean Ct value 14.78 ± 3.59) is the most abundantly expressed gene (i.e., lowest average Ct value), whereas *GAPDH* (mean Ct value 31.43 ± 4.02) showed the lowest expression (i.e., highest average Ct value).

### 2.3. Gene Expression Stability Comparative *Δ*Ct and BestKeeper

**Figure 5:**
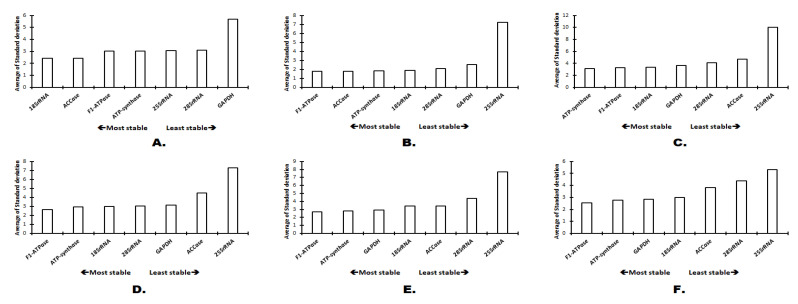
Evaluation of expression stability by ΔCt method: (**A**). Plant organs; (**B**) Osmotic stress; (**C**) Salt stress; (**D**) Elicitor stress; (**E**) Temperature stress; (**F**) All combined.

**Figure 6:**
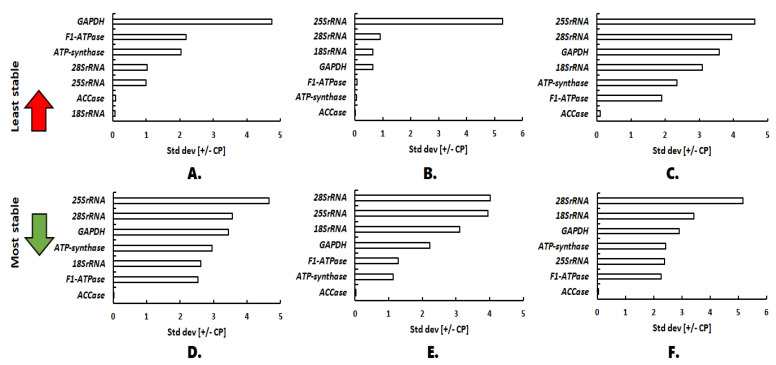
Evaluation of expression stability by BestKeeper: (**A**) Plant organs; (**B**) Osmotic stress; (**C**) Salt stress; (**D**) Elicitor stress; (**E**) Temperature stress; (**F**) All combined.

The method ΔCt evaluates the stability of the candidate reference gene expression based on the standard deviation values whereas the BestKeeper software determines the consistency of the reference genes based on the CP and standard deviation (SD) values. In the case of expression studies with the plant organs (callus, stem and root), both ΔCt and BestKeeper identified *18S rRNA* as the best candidate for reference gene (Figure 5 and Figure 6). Under all the other experimental conditions, *ACCase* was observed to be the most stable reference gene, according to BestKeeper (Figure 6). However, ΔCt suggested *ATP-synthase* as the best reference gene under salt stress and *F1-ATPase* under osmotic stress, elicitor stress, temperature stress and under combined conditions (Figure 5).

### 2.4. Gene Expression Stability Using NormFinder

NormFinder software assessed the intra- and intergroup variations, which were then combined into stability values. Finally, the candidate gene with the least variation was ranked as the best by the software. Additionally, this software also recommended the best combination of genes using a pairwise comparison approach. In this study, *ATP-synthase* has been considered to be the most stable candidate under osmotic stress, salt stress and combined condition, whereas *25S rRNA* was observed to be the best candidate for the expression studies with plant organs and under elicitor and temperature stress. *GAPDH* was considered to be the least stable under all conditions. NormFinder algorithm also identified the best pair of genes. Table 2 shows the most stable reference gene as well as the best combination in rosemary, as evaluated by NormFinder.

### 2.5. Gene Expression Stability Using geNorm

**Figure 7:**
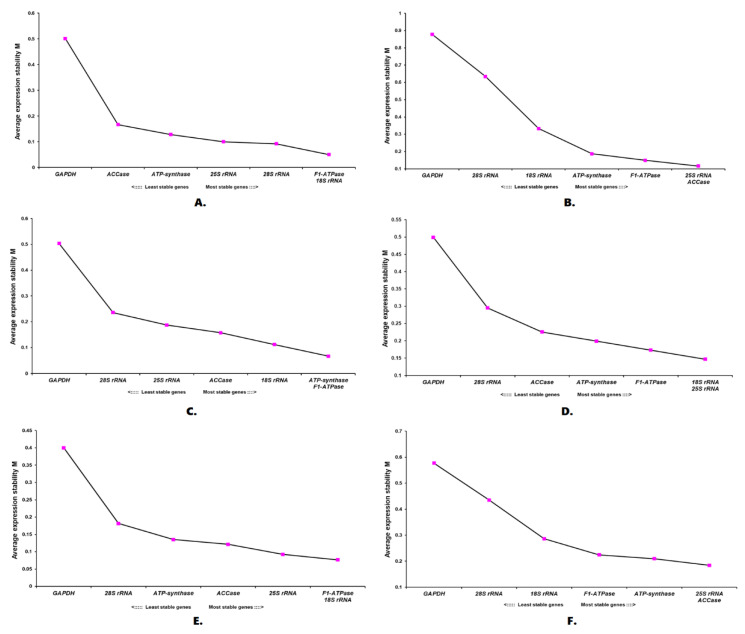
Evaluation of expression stability by geNorm: (**A**) Plant organs; (**B**) Osmotic stress; (**C**) Salt stress; (**D**) Elicitor stress; (**E**) Temperature stress; (**F**) All combined.

**Figure 8:**
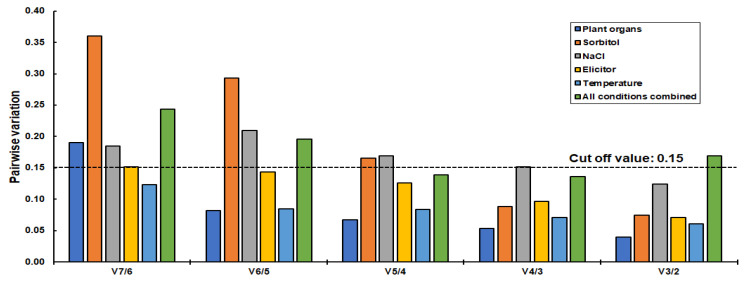
Analysis of the number of optimal genes required for each experimental condition.

The geNorm software algorithm assessed the stability of the reference genes based on the geometric means values or “M” values. This software considered 1.5 as the threshold value. The genes with an M value <1.5, were deemed to be the most stable reference gene candidate. In addition to the reference gene expression stability, geNorm was also used to calculate the pairwise variation, based on which the optimal number of reference gene/s required for a particular experimental condition is/are calculated. The results of the gene expression stability analysis with geNorm are shown in Figure 7. In our study, geNorm identified *F1-ATPase*/*18S rRNA* as the best combination for studies with plant organs and under temperature stress. The gene pairs, *ATP-synthase*/*F1-ATPase* and *18S rRNA*/*25S rRNA* were the recommended genes under salt and elicitor stress, respectively. geNorm ranked *25S rRNA*/*ACCase* as the best reference gene combination under osmotic stress as well as under combined conditions. Irrespective of the experimental conditions, *GAPDH* performed poorly, and hence, is not recommended for gene expression normalization by geNorm software. Based on the pairwise analysis, two reference genes are sufficient for the normalization of RT-qPCR data under all conditions except combined conditions (Figure 8). Given that two reference genes are sufficient, the gene pairs identified by geNorm should be considered. Although, in combined conditions, a combination of three genes is recommended by geNorm (*25S rRNA*, *ACCase*, and *ATP-synthase*).

### 2.6. Gene Expression Stability Using RefFinder

To remove the heterogeneity in ranking order (provide by all the mentioned software), RefFinder is used to produce a comprehensive ranking of the candidate reference genes (Table 3). This software integrated the results obtained from geNorm, NormFinder, BestKeeper, and the comparative ΔCt method to produce an overall grading of the most stable reference genes. In this study, for gene experiments with plant organs, RefFinder recommends using *18S rRNA* as the most stable reference gene. *GAPDH* was considered the least stable gene. *ATP-synthase* and *25S rRNA* was considered the best and the worst reference gene, respectively, under the salt and temperature stress conditions. Under osmotic stress, *ACCase* and *25S rRNA* were observed as the most and least stable candidates, respectively. Under elicitor stress and the combined condition, RefFinder recommended *F1-ATPase*, as the most stable reference gene.

### 2.7. Choice of the Best Reference Gene and Validation under Nanoparticle Stress

**Figure 9:**
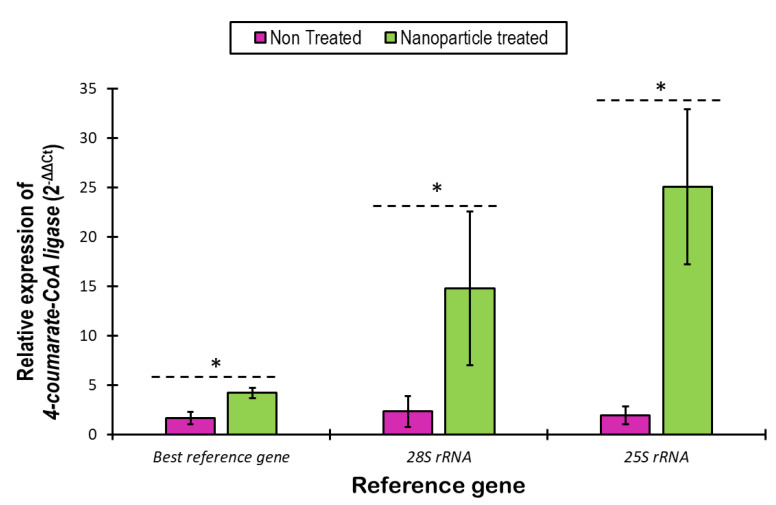
Relative expression of the *4-coumarate-CoA ligase* gene under zinc nanoparticle stress. Relative gene expression without any nanoparticle treatment and 7 days after zinc nanoparticle treatment were compared, and normalization was performed with the best reference gene (combination of *F1-ATPase*, *ATP synthase* and *ACCase*) and the worst reference genes (*28S rRNA* and *25S rRNA*). The (*) indicates significance at a 5% significance level.

Based on the results of the pairwise variation, using the combination of *F1-ATPase*/*ATP synthase*/*ACCase* for normalization of the gene expression studies in rosemary produced by plant tissue culture methods is recommended. Moreover, we also endorse not using *25S rRNA* and *28S rRNA* for RT-qPCR studies with in vitro *S. rosmarinus*. For all individual conditions, pairwise variation results suggest a combination of *F1-ATPase*/*ATP synthase* gene pair as the best reference genes, except for plant organ and osmotic stress where gene pairs *18S rRNA*/*ACCase* and *ACCase*/*F1-ATPase,* respectively, are recommended. Further, to authenticate the trustworthiness of the candidate genes, the relative expression of the *4CL* under nanoparticle stress was assessed using the best and the least stable candidate genes. The normalized relative expression of *4CL* (by the best and the worst gene) in *S. rosmarinus* plants is shown in Figure 9. In all the cases, the relative expression of the *4CL* is higher following the nanoparticle treatment, however, while normalization using *25S rRNA* and *28S rRNA*, atypical overexpression was detected. In the case of normalization using the best pair, we found ~2.5× overexpression of *4CL*, following the nanoparticle treatment. However, in the other cases, ~6.3× (for *28S rRNA*) and ~13× (for *25S rRNA*) overexpression of *4CL* was detected, following the nanoparticle treatment.

## 3. Discussion

Plant tissue culture or in vitro systems offer several advantages over field conditions such as: extraction of metabolites is independent of environmental factors and growing seasons; any plant or plant cells can be multiplied targeting specific metabolites; elicitors and hormones can be efficiently employed; organic substances could be extracted which cannot be achieved by chemical synthesis. To achieve these, researchers have utilized several approaches such as callus culture and bioreactor culture along with several biotic and abiotic stresses, elicitor, and nanoparticle treatment [21,22,23]. However, the underlying molecular mechanisms behind this secondary metabolite production are yet to be explored and need to be studied extensively, which will require RT-qPCR experiments. RT-qPCR has the ability to detect very low-level expression of genes providing it an edge over other traditional methods. However, the accuracy of this method is highly dependent on the selection of stable reference gene/s for the normalization process. Earlier, reference genes were believed to be stable in all given conditions and were utilized for the normalization process however, recent findings have reported that the expression of these genes could vary considerably, even the classical reference genes [24,25]. Given that, identification of reference gene/s for normalization in any given experimental condition is very crucial, if not optimized properly might lead to ambiguous outcomes.

Considering the industrial importance of rosemary, it is surprising that there had not yet been any validated reference gene reported for in vitro-produced *S. rosmarinus*. Hence, in this study, we used RT-qPCR to evaluate the expression stability of seven candidate reference genes under various experimental conditions. The minimum number of required reference genes for each experimental condition was also identified. Additionally, the most stable reference gene/s for each experimental condition were recommended, individually. Based on the pairwise analysis, we suggest using a combination of *F1-ATPase*/*ATP-synthase* for gene expression studies under salt stress, elicitor stress, and temperature stress. For plant organ and osmotic stress, we recommend gene pairs *18S rRNA*/*ACCase* and *ACCase*/*F1-ATPase*, respectively. However, considering all the experimental conditions, we also recommend using a combination of *F1-ATPase*/*ATP synthase*/*ACCase* for the normalization of gene expression studies in rosemary produced under plant tissue culture.

Although we had identified and validated reference genes suitable for normalizing RT-qPCR experiments in in vitro-produced rosemary, there are no internal reference genes that are stably expressed under all experimental conditions. For example, in a previous study with *Rosmarinus officinalis*, the authors recommend using *GAPDH* for normalization of the RT-qPCR data [13], but the current study suggests that this gene may be unsuitable for expression studies with plant tissue culture-produced rosemary. These contradictory differences might be attributed to plant tissue selection, different stress treatments and experimental treatments carried out under in vitro conditions. Similarly, a study aiming to screen out suitable reference genes in two thyme species (Lamiaceae family), recommended *GAPDH* and *18S rRNA* as the best and the worst genes, respectively [24]. Much research has also demonstrated that the expression levels of reference genes might vary not only within tissues or development stages but also even get influenced by various biotic and abiotic stresses [7,26]. For example, a study with genome-wide identification and assessment of novel reference genes in wheat identified stable expression for *ACT* and *UBQ*, but not in tomato plants [27,28]. Whereas, in another study, *GAPDH* is highly recommended for gene expression studies in grape berries, but not in wheat [27,29]. Similarly, a previous study showed that in *Salvia hispanica* under various abiotic stresses, *GAPDH* appeared to be the best candidate [30]. In contrast, *GAPDH* was considered to be an inappropriate gene for normalization in strawberries (under drought and salt stress) [31], *Zea mays* (under flowering and water deficit stress) [32]. Our study also does not recommend using *GAPDH* in rosemary produced under in vitro conditions.

Understanding how plant and plant cells interact and perceive various treatments and elicitors will provide valuable insights to further enhance metabolite production in industrially important plants such as rosemary. Rapid progress in molecular biology methods in plant biotechnology has increased the demand for the identification of reference genes. Additionally, with a significant decrease in the cost of the comparative RNA-seq transcriptome analysis, suitable reference genes are needed for the validation of the RNA-seq results. This is the first study to evaluate and authenticate experiment-condition-specific reference genes in rosemary produced in vitro, to the best of our knowledge. Hence, the reference genes recommended in our study will aid in the studies related to the elucidation of abiotic stresses and their regulatory mechanisms.

## 4. Materials and Methods

### 4.1. Plant Material Acquisition, Surface Sterilization, Preparation of Culture Medium and Growth Conditions

Explants of rosemary were taken from the well-maintained botanical garden of the Faculty of Tropical AgriSciences (FTZ), Czech University of Life Sciences, Prague. No specific permissions were necessary to obtain the plant samples. All experiments conducted in the current study, are in compliance with relevant institutional, national, and international guidelines and legislation. Thereafter, the explants were rinsed with distilled water for 10 min. Following the rinsing, the explants were treated with 70% ethanol (*v*/*v*) for 2 min, then 1% solution of sodium hypochlorite (*v*/*v*) (NaOCl, commercial bleach-SAVO) containing two drops of Tween 20 for 10 min. Finally, explants were rinsed with autoclaved distilled water three times, then explants were transferred to Murashige and Skoog’s (1962) (MS) basal medium under a laminar flow hood for initiation. The pH of the medium was adjusted to 5.7 ± 1, solidified with 8 g/L agar, and sterilized in an autoclave (Nuve 0T-032 model) at 121 ℃ for 20 min. Inoculated cultures were maintained at 24/20 ± 1 ℃ (except for treatments: heat and cold), 16/8h (light/dark) photoperiod, and relative humidity of 60–70%.

### 4.2. Callus Induction

Surface sterilized leaf explants were utilized for callus induction. Different combinations of 6-benzylaminopurine (BAP), 2,4-Dichlorophenoxyacetic acid (2,4-D), and Kinetin (Kn) (Table 4) were used to test optimum concentration and combination to induce callus.

### 4.3. Stress, Elicitor, and Nanoparticle Treatment

The in vitro-grown plants and callus of *S. rosmarinus* were subjected to different stresses and elicitors supplemented with the basic MS. The treatments included: Osmotic stress (Sorbitol, 200mM), Salt stress (NaCl, 100mM), Heat stress (30 ℃), Cold stress (4 ℃), Jasmonic acid (1 mg/L), Casein hydrolysate (1 mg/L). Additionally, ZnO (Zinc Oxide) NPs at a concentration of 10 mg/L were used for nanoparticle stress. ZnO NPs nanopowder, <50 nm particle size (BET), >97% SIGMA-ALDRICH (677450-5G) were obtained from Sigma-Aldrich (Burlington, MA, USA). To disrupt and dissolve the ZnO NPs, they were dispersed in sterile distilled water for 60 min using a sonicator (BANDALIN SONOPLUS). After that, the solution was further diluted to prepare desired concentrations. All the treatments had five replications and were maintained for 24 h except for nanoparticle treatment which was maintained for 7 days. Plant samples from leaves and stems and callus were collected from each treatment to be stored at −80 °C (until further use).

### 4.4. RT-qPCR Experiment

The plant RNA was extracted from the fresh tissues (± 60–80 mg per sample) using the RNeasy Mini Kit (Qiagen, Hilden, Germany). The RNA sample integrity was confirmed by running the samples on 1.2% agarose gel electrophoresis and the quality of the RNA was checked by NanoDrop spectrophotometer (NanoDrop™ 2000/2000c Spectrophotometer, Thermo Scientific™, Waltham, MA, USA). A total of 1 μg (per reaction) of quality-checked gDNA-free RNA template was used as a template for cDNA synthesis. TURBO DNA-free™ (Invitrogen, Waltham, MA, USA) Kit was used for gDNA removal according to the manufacturer’s instructions. Further, the cDNA synthesis was performed by a High-Capacity cDNA Reverse Transcription Kit (Thermo Fisher Scientific, USA), according to the manufacturer’s instructions. Gene-specific primers were designed for seven common candidate reference genes (*18S rRNA*, *25S rRNA*, *28S rRNA*, *ACCase*, *GAPDH*, *ATP-synthase* and *F1-ATPase*). The candidate genes were chosen based on previous literature studies and the primers were designed based on their homologous sequences in related or other plant species using Primer3 software (https://bioinfo.ut.ee/primer3-0.4.0/ (accessed on 21 March 2022)). All the designed primers were tested by general PCR (C1000 thermocycler, Bio-Rad, Hercules, CA, USA) and verified in the 1.8% agarose gel electrophoresis. The RT-qPCR assay was performed in StepOne™ Real-Time PCR System (Applied Biosystems™, Waltham, MA, USA) using PowerUp™ SYBR™ Green Master Mix (Applied Biosystems™, USA). The reaction mixture used was as follows: 5 μL of SYBR Green Master Mix, 1 μL of primer mix (10 mM each), and 4 μL of cDNA (2.5 ng/μL). The primer efficiency (E) and correlation coefficient (R2) calculation was performed according to a previous study^8^. The RT-qPCR thermocycler was programmed at an initial denaturation step at 95 °C for 10 min, followed by 40 cycles of 15 s at 95 °C and 1 min at 57 to 62 °C (based on the annealing temperature of the primer pairs). For melting curve analysis, stepwise heating was performed from 60 °C to 95 °C. Five biological replicates were used for all the RT-qPCR experiments.

### 4.5. Reference Gene Analysis

Gene expression stabilities of the candidate genes were examined by comparative ΔCt, RefFinder, geNorm, NormFinder and BestKeeper, according to previous literature [8]. Before and after nanoparticle stress treatment, the relative *4CL* gene expression was calculated using the 2^-ΔΔCt^ method [33]. NormFinder software ranked the gene based on the stability values and the gene with minimal variation was ranked as the best by the software. geNorm program estimated the ranking of the candidate genes based on the expression stability value (M). The M value was inversely proportional to the ranking of the gene. BestKeeper relied on the standard deviation values of cycle threshold (Ct) or crossing point values (CP) and coefficient of correlation (r) values. RefFinder integrates all the software for reference gene screening (geNorm, NormFinder, BestKeeper, and the comparative ΔCt method) and gave a complete ranking based on the geometric mean of ranking values [24].

### 4.6. Statistical Analysis

The results for relative *4CL* expression were compared using a two-sample t-test in Microsoft Excel 2021 with XLSTAT (version 2022.1, Microsoft, Redmond, DC, USA) (https://www.xlstat.com/en/ (accessed on 10 May 2022)). For this purpose, we used the average Ct value from five biological replicates and the relative gene expression levels were calculated using the 2^−△△Ct^ method.

## 5. Conclusions

To achieve accurate results for gene expression studies by RT-qPCR, the selection of suitable reference genes is a prerequisite. In the current study, a set of seven genes were systematically examined for their suitability for gene expression normalization in *S. rosmarinus* under various in vitro experimental treatments and conditions. Based on the results, we recommend using a combination of *F1-ATPase*/*ATP synthase*/*ACCase* to normalize the gene expression experiments in *S. rosmarinus* under in vitro conditions. Additionally, we also discourage the use of *25S rRNA* and *28S rRNA* for RT-qPCR studies with in vitro *S. rosmarinus*. To the best of our knowledge, the current study is the first to validate reference genes in *S. rosmarinus* produced under in vitro conditions for RT-qPCR data normalization. The findings of the current study lay a foundation for the studies aiming to explore the underlying molecular mechanisms in *S. rosmarinus* produced under in vitro conditions with various stress and elicitors.

## Figures and Tables

**Table 1:** Effect of different treatments of phytohormones on callus induction (%) of *Salvia rosmarinus*.

Treatment	Callus Induction% (± Standard Deviation)	Growth Status of Callus
C1	100 ± 0.0	Proliferated fast, Light green to dark green in color and structurally friable
C2	100 ± 0.0	Proliferated fast, light green in color and structurally friable
C3	100 ± 0.0	Proliferated slowly, light green to brown in color and structurally friable
C4	100 ± 0.0	Proliferated slowly, brown color and structurally friable
C5	100 ± 0.0	Proliferated slowly, brown in color and structurally friable
C6	100 ± 0.0	Proliferated slowly, brown in color and structurally friable

**Table 2:** Evaluation of expression stability by NormFinder.

**Rank**	**Plant Organ**	**Osmotic stress**	**Salt STRESS**
1	*25S rRNA*	*ATP-synthase*	*ATP-synthase*
2	*28S rRNA*	*25S rRNA*	*F1-ATPase*
3	*18S rRNA*	*ACCase*	*18S rRNA*
4	*ATP-synthase*	*F1-ATPase*	*25S rRNA*
5	*F1-ATPase*	*18S rRNA*	*ACCase*
6	*ACCase*	*28S rRNA*	*28S rRNA*
7	*GAPDH*	*GAPDH*	*GAPDH*
Best pair	*25S rRNA* */28S rRNA*	*ATP-synthase* */25S rRNA*	*ATP-synthase* */F1-ATPase*
**Rank**	**Elicitor stress**	**Temperature stress**	**All combined**
1	*25S rRNA*	*25S rRNA*	*ATP-synthase*
2	*F1-ATPase*	*18S rRNA*	*25S rRNA*
3	*18S rRNA*	*F1-ATPase*	*F1-ATPase*
4	*ATP-synthase*	*ATP-synthase*	*18S rRNA*
5	*ACCase*	*ACCase*	*ACCase*
6	*28S rRNA*	*28S rRNA*	*28S rRNA*
7	*GAPDH*	*GAPDH*	*GAPDH*
Best pair	*F1-ATPase* */25S rRNA*	*F1-ATPase* */25S rRNA*	*F1-ATPase* */18S rRNA*

**Table 3:** Evaluation of expression stability by RefFinder.

**Rank**	**Plant Organ**	**Osmotic stress**	**Salt Stress**
1	*18S rRNA*	*ACCase*	*ATP-synthase*
2	*ACCase*	*F1-ATPase*	*F1-ATPase*
3	*25S rRNA*	*18S rRNA*	*GAPDH*
4	*28S rRNA*	*ATP-synthase*	*ACCase*
5	*ATP-synthase*	*28S rRNA*	*18S rRNA*
6	*F1-ATPase*	*GAPDH*	*28S rRNA*
7	*GAPDH*	*25S rRNA*	*25S rRNA*
**Rank**	**Elicitor stress**	**Temperature stress**	**All combined**
1	*F1-ATPase*	*ATP-synthase*	*F1-ATPase*
2	*ATP-synthase*	*F1-ATPase*	*ATP-synthase*
3	*18S rRNA*	*ACCase*	*ACCase*
4	*ACCase*	*GAPDH*	*GAPDH*
5	*28S rRNA*	*18S rRNA*	*18S rRNA*
6	*GAPDH*	*28S rRNA*	*25S rRNA*
7	*25S rRNA*	*25S rRNA*	*28S rRNA*

**Table 4:** Combinations of phytohormones utilized for callus induction in *Salvia rosmarinus*.

Combinations	BAP (mg/L)	2,4-D (mg/L)	Kinetin (mg/L)
C1	1.5	0.5	-
C2	1.0	1.0	-
C3	0.5	1.5	-
C4	-	1.5	0.5
C5	-	1.0	1.0
C6	-	0.5	1.5

## Data Availability

Data is contained within the article or supplementary material.

## Supplementary Materials

The following supporting information can be downloaded at: https://www.mdpi.com/article/10.3390/plants11212878/s1, Table S1: Primer efficiency and correlation coefficient values of the candidate reference genes.
