# Supplementary material for: Selection and Validation of the Most Suitable Reference Genes for Quantitative Real-Time PCR Normalization in Salvia rosmarinus under In Vitro Conditions
Source: Plants (Basel). 2022 Oct 27;11(21):2878. doi: 10.3390/plants11212878 (PMC9655146; doi:10.3390/plants11212878)
Supplement: Supplementary file 1 [file plants-11-02878-s001.zip › Supplementary.pdf]

Selection and validation of the most suitable reference genes for quantitative real-time PCR normalization in *Salvia rosmarinus* under *in vitro* conditions

Rohit Bharati[1], Madhab Kumar Sen[2], Ram Kumar[3], Aayushi Gupta[4], Vishma Pratap Sur[5], Ingrid Melnikovová[1], Eloy Fernández-Cusimamani[1*]

Short title: Reference genes for molecular studies in rosemary

[1]Department of Crop Sciences and Agroforestry, The Faculty of Tropical AgriSciences, Czech University of Life Sciences Prague, Kamýcká 129, 165 00 Prague 6, Suchdol, Czech Republic.

[2]Department of Agroecology and Crop Production, Faculty of Agrobiology, Food and Natural Resources, Czech University of Life Sciences Prague, Kamýcká 129, 165 00 Prague 6, Suchdol, Czech Republic.

[3]Department of Plant Protection, Faculty of Agrobiology, Food and Natural Resources, Czech University of Life Sciences Prague, Kamýcká 129, 165 00 Prague 6, Suchdol, Czech Republic.

[4]Department of Botany and Plant Physiology, Faculty of Agrobiology, Food and Natural Resources, Czech University of Life Sciences Prague, Kamýcká 129, 165 00 Prague 6, Suchdol, Czech Republic.

[5]Laboratory of Reproductive Biology, Institute of Biotechnology of the Czech Academy of Sciences, BIOCEV, Prumyslova 595, 252 50 Vestec, Czech Republic.

***Address correspondence to**:

Eloy Fernández-Cusimamani

Department of Crop Sciences and Agroforestry, The Faculty of Tropical AgriSciences, Czech University of Life Sciences Prague, Kamýcká 129, 165 00 Prague 6, Suchdol, Czech Republic. Email: eloy@ftz.czu.cz

Table S1: Primer efficiency and correlation coefficient values of the candidate reference genes.

Gene specific primers	GenBank accessions used	Sequence	Annealing temperature (°C)	Amplicon length (bp)	Primer efficiency (%)	R^2 value
18S rRNA_Fwd	X16077.1, AH001709.2, XR_004642731.1	TCTGCCCTATCAACTTTCGATGGTA	62	168	93.90	0.99
18S rRNA_Rev		AATTTGCGCGCCTGCTGCCTTCCTT				
28S rRNA_Fwd	XR_006991982.1, AH001710.2, XR_006995432.1, XR_006614521.1	CCTGATCTTCTGTGAAGGGTTCGA	60	95	93.93	0.99
28S rRNA_Rev		GGTTCGATTAGTCTTTCGCCCCTA				
25S rRNA_Fwd	NC_056052.1 X13557.1; NR_137326.1	TGGATCTGGGACTGCTCTTG	57	182	104.69	1.00
25S rRNA_Rev		AGGACCCTCTTCTGGAAAGC				
ATP-Synthase_Fwd	NM_114510.3, OM691673.1, NC_061230.1	GGCTTGAACGAAACGGAAGA	59.5	115	101.13	0.99
ATP-Synthase_Rev		AGAGTTGGTTTGACTGCCCT				
F1-ATPase_Fwd	D88375.1	TATCTGTCAGTCGTGTCGGG	59.1	110	98.84	0.99
F1-ATPase_Rev		AAAGGCGGCTACTTCTCGAT				
GAPDH_Fwd	KX086568.1	GGACTGGAGAGGTGGAAGAG	60.1	135	100.92	1.00
GAPDH_Rev		GGAACCCTGAATGACATGCC				
ACCase_Fwd	E09394.1	GCTGCTATTGCCAGTGCTTA	57	53	93.87	1.00
ACCase_Rev		AAGCTTGTTCAGGGCAGAAA				
